# Supplementary material for: Ist1 regulates ESCRT-III assembly and function during multivesicular endosome biogenesis in Caenorhabditis elegans embryos
Source: Nat Commun. 2017 Nov 13;8:1439. doi: 10.1038/s41467-017-01636-8 (PMC5682282; doi:10.1038/s41467-017-01636-8)

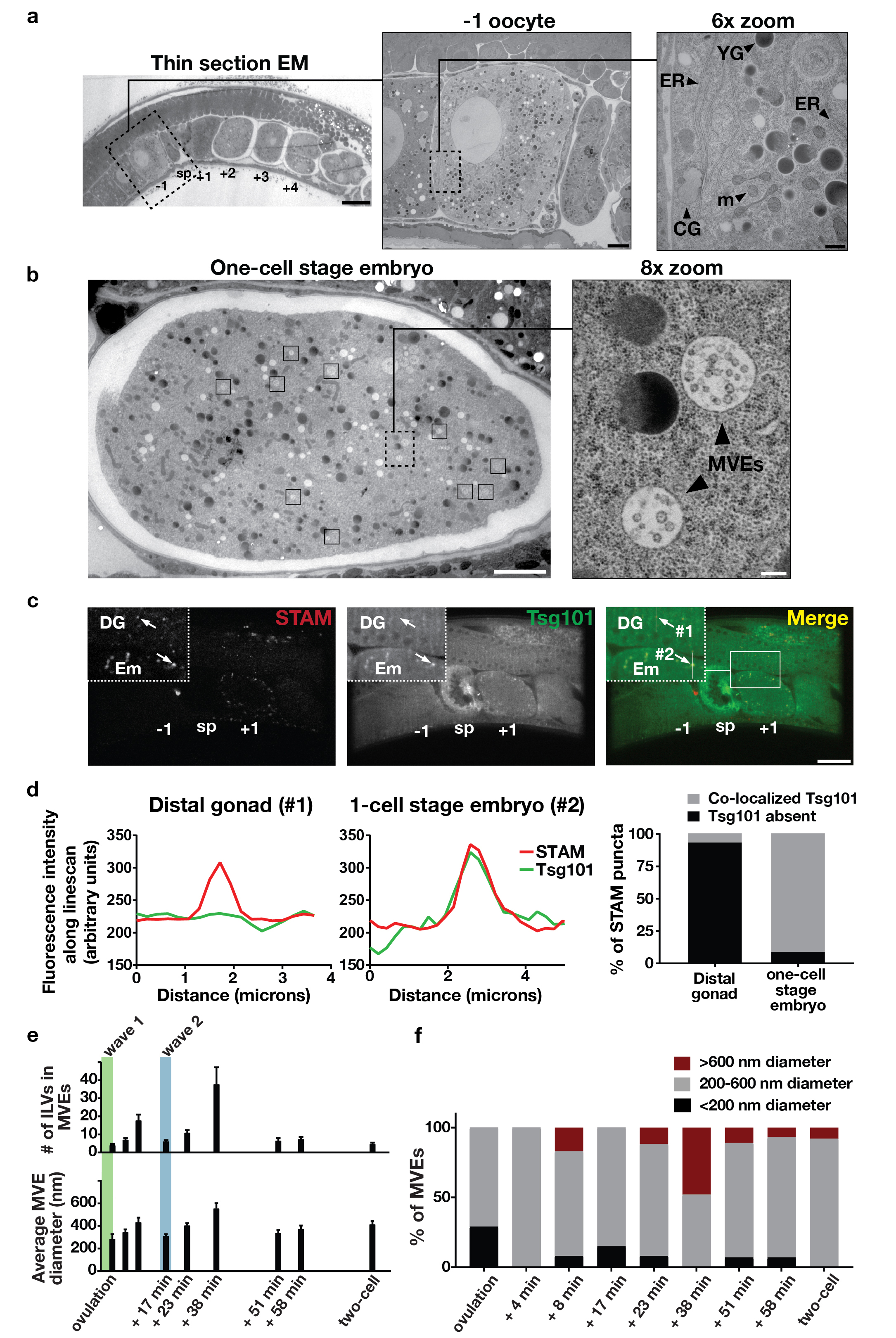


**Supplementary Figure 1.** **The ESCRT machinery acts to generate MVEs *de novo* following oocyte fertilization and ovulation.** (A) Animals were high pressure frozen and processed for thin sectioning and electron microscopy. 20 oocytes were analyzed to confirm the absence of MVEs, and representative images are shown. Endoplasmic reticulum (ER), cortical granules (CG), mitochondria (m), and yolk granules (YG) are highlighted. Bars, 25 μm (left panel), 4 μm (middle panel) and 600 nm (right panel). (B) Animals were high pressure frozen and processed for thin sectioning and electron microscopy. One-cell embryos consistently contained multiple MVEs (left panel, boxed regions). 30 one-cell stage embryos were analyzed to confirm the presence of MVEs. Bars, 5 μm (left panel) and 100 nm (right panel). (C) Animals co-expressing mCherry::STAM and GFP::Tsg101 were imaged using confocal microscopy (n=10 animals). The presence of each was detected in the distal gonad (DG) and embryos (Em). Arrows indicate the positions where linescan measurements were made. Representative images are shown. Bar, 20 μm. (D) Linescan analysis showing the fluorescence intensities of mCherry::STAM and GFP::Tsg101 along lines highlighted in panel C. Quantification of the frequency of STAM and Tsg101 co-localization in the distal gonad (DG) and early embryo (Em) is shown (right). 10 animals were analyzed for each condition. (E) The number of ILVs within MVEs and the average diameter of MVEs are shown relative to embryo development. The timing of the two waves of endocytosis that occurs after oocyte fertilization is highlighted. At each timepoint, 10 MVEs were analyzed. Each error bar represents standard error of the mean. (F) The relative distribution of MVE diameters during development of the one-cell stage embryo is shown, based on the analysis of 10 MVEs at each timepoint.


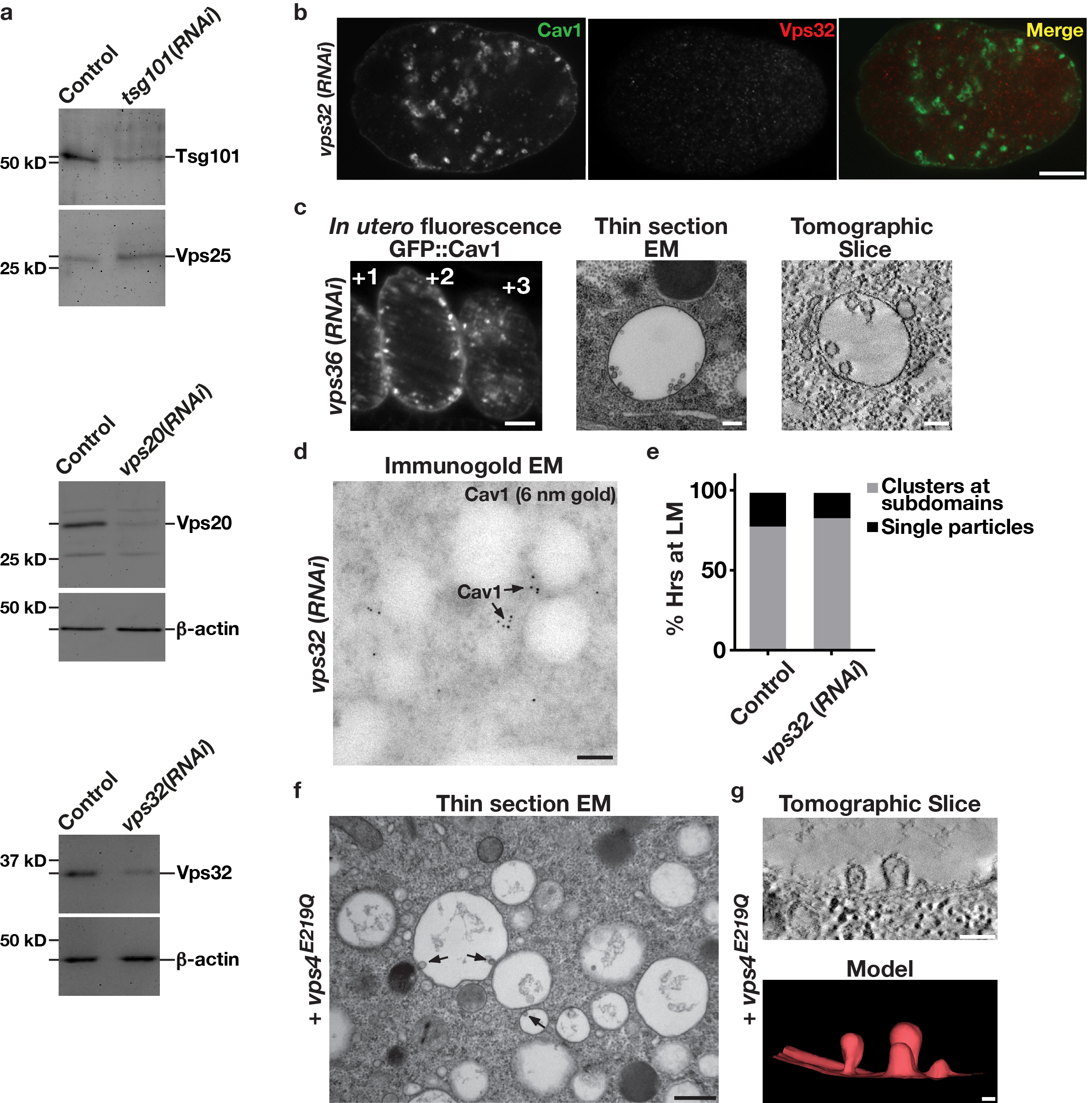


**Supplementary Figure 2. Perturbation of ESCRT function inhibits MVE biogenesis.** (A) Extracts from control and ESCRT subunit depleted animals were separated by SDS-PAGE and immunoblotted with the indicated antibodies to confirm depletion efficiency (n=3 each). (B) Embryos expressing GFP::Cav1 and depleted of Vps32 were fixed and stained using antibodies directed against GFP and Vps32. Images are representative of 20 embryos treated similarly. Bar, 10 μm. (C) Animals expressing GFP::Cav1 and depleted of Vps36 (a core subunit of ESCRT-II) were imaged using fluorescence and electron microscopy. Images are representative of 15 animals imaged using fluorescence microscopy and 30 MVEs analyzed by thin section electron microscopy and electron tomography. Bars, 10 μm (left, fluorescence) and 100 nm (middle and right panels). (D) Immunogold EM (6 nm gold directed against GFP) was used to measure the distribution of GFP::Cav1 on small endosomes (120-250 nm in diameter) that accumulate following depletion of Vps32 (n=24). A representative image is shown. Bar, 100 nm. (E) The distribution of Hrs at the limiting membrane (LM) of endosomes was measured in control (n=35) and Vps32 depleted embryos (n=36). Clusters were defined as the presence of 2 or more gold particles less than 20 nm apart. (F) Animals expressing Vps4^E219Q^ were high pressure frozen and processed for thin section electron microscopy. Arrows highlight nascent ILVs that accumulate under this condition (n=10 animals). A representative image is shown. Bar, 500 nm. (G) Animals expressing Vps4^E219Q^ were high pressure frozen and processed for electron tomography (n=8 animals). Both a representative tomographic slice (top panel) and a representative reconstructed model (bottom panel) are shown. Bars, 100 nm (top panel) and 25 nm (bottom panel).





**Supplementary Figure 3. Ist1 functions in MVE biogenesis but not endocytic recycling in *C. elegans*.** (A) Cartoon highlighting the similar domain architecture of *C. elegans* Ist1 and human Ist1. Amino terminal alpha helices and carboxyl-terminal microtubule interacting motifs (MIMs) are specified. The *tm7401* lesion is indicated, introducing a frameshift and truncating the last 84 amino acids from *C. elegans* Ist1, including both MIM domains. Additionally, a peptide encoded by an isoform 1-specific exon is highlighted in the *C. elegans* protein. (B) Mutant animals expressing GFP::Cav1 and harboring the *tm7401* deletion were imaged using confocal microscopy. The image is representative of 20 animals analyzed. Bar, 10 μm. (C) Animals expressing Mig14::GFP were imaged using confocal microscopy in the presence or absence of Vps35 (a core subunit of the retromer complex) or Ist1. Regions where linescan measurements were made are indicated (at the junction between the ABa and ABb cells). Data are representative of 10 animals examined. Bars, 10 μm. (D) Linescan analysis of Mig14::GFP fluorescence intensity at cell-cell junctions highlighted in panel C.


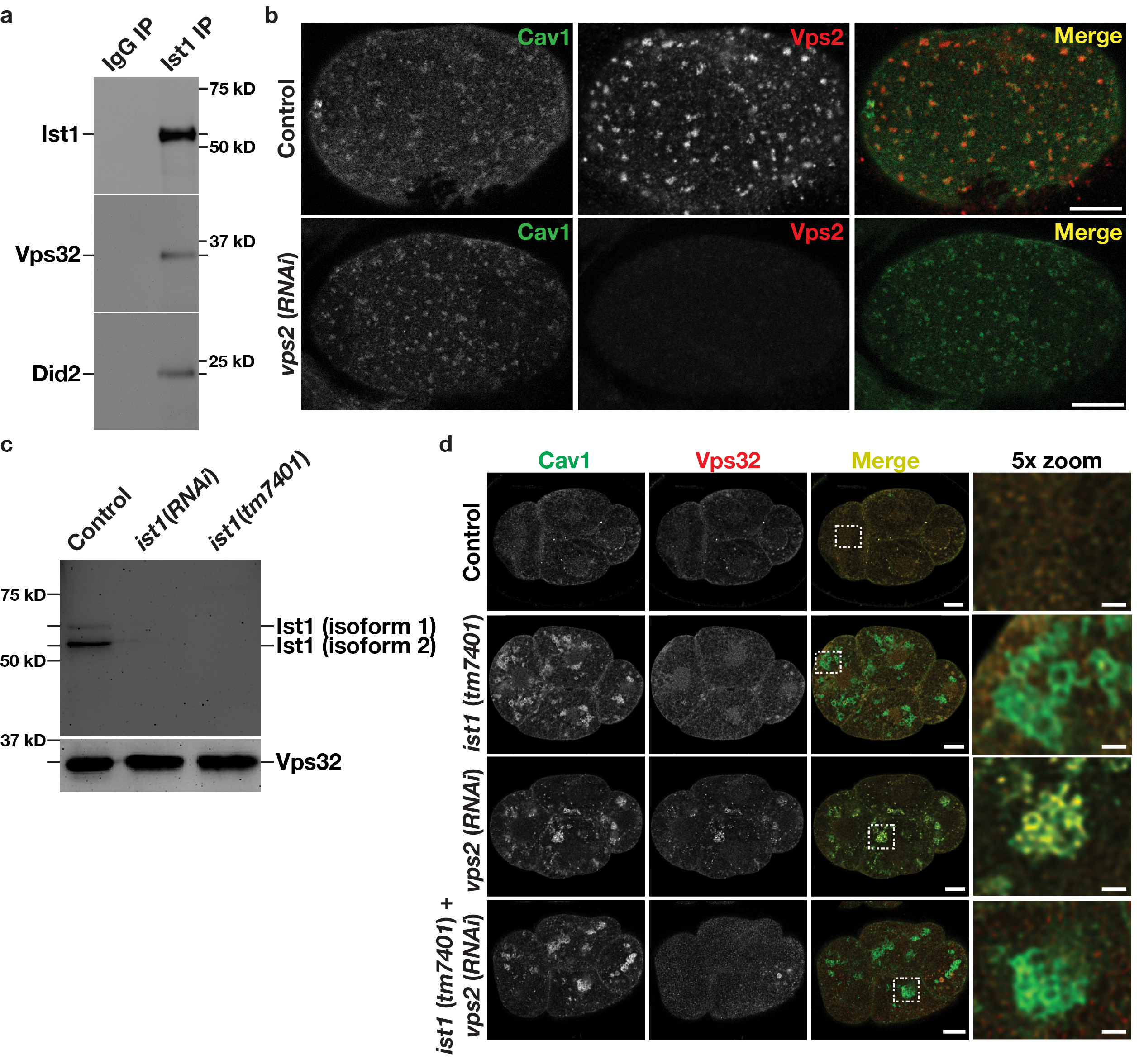


**Supplementary Figure 4. Depletion of Ist1 impairs Vps32 assembly at endosomes.** (A) Extracts from control embryos were immunoprecipitated using rabbit IgG or antibodies directed against Ist1 and immunoblotted using the antibodies shown following their separation by SDS-PAGE (n=3 each). (B) Embryos expressing GFP::Cav1 in the presence or absence of Vps2 were fixed and stained using antibodies directed against GFP and Vps2. Images are representative of 10 embryos treated similarly. Bars, 10 μm. (C) Extracts from control animals, animals depleted of Ist1, or animals harboring the *tm7401* deletion mutation were separated by SDS-PAGE and immunoblotted for Ist1 and Vps32 (n=3 each). (D) Control (n=5) and *ist1* (*tm7401*) mutant embryos (n=5) expressing GFP::Cav1 and depleted of Vps2 using RNAi were fixed and stained using antibodies directed against GFP and Vps32. Samples were imaged using STED microscopy, and representative images are shown. Bars, 10 μm (left panels) and 1 μm (right, zoomed panels).

**
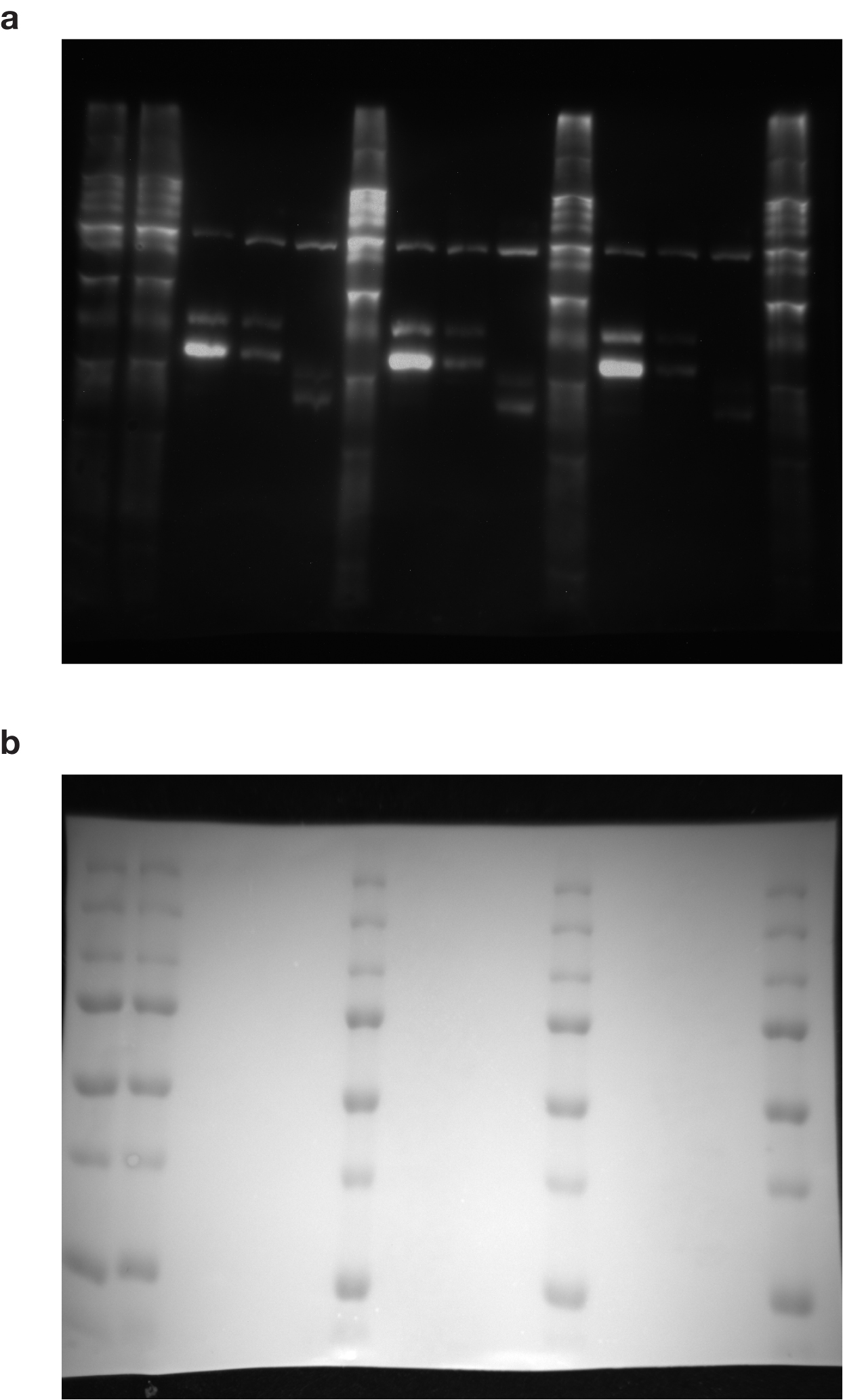
**

**Supplementary Figure 5. Uncropped scans of the immunoblot shown in the main set of figure panels.** (A) Uncropped and unprocessed digital scan of the immunoblot shown in Fig. 4b. The immunoblot was performed in triplicate and imaged on an Epi Chemi II Darkroom imager (UVP Laboratory Products). (B) Brightfield image of the nitrocellulose paper used in panel A for immunoblotting.

**Supplementary Table 1. Identification of interactions between Ist1 and other ESCRT-III subunits using solution mass spectrometry**


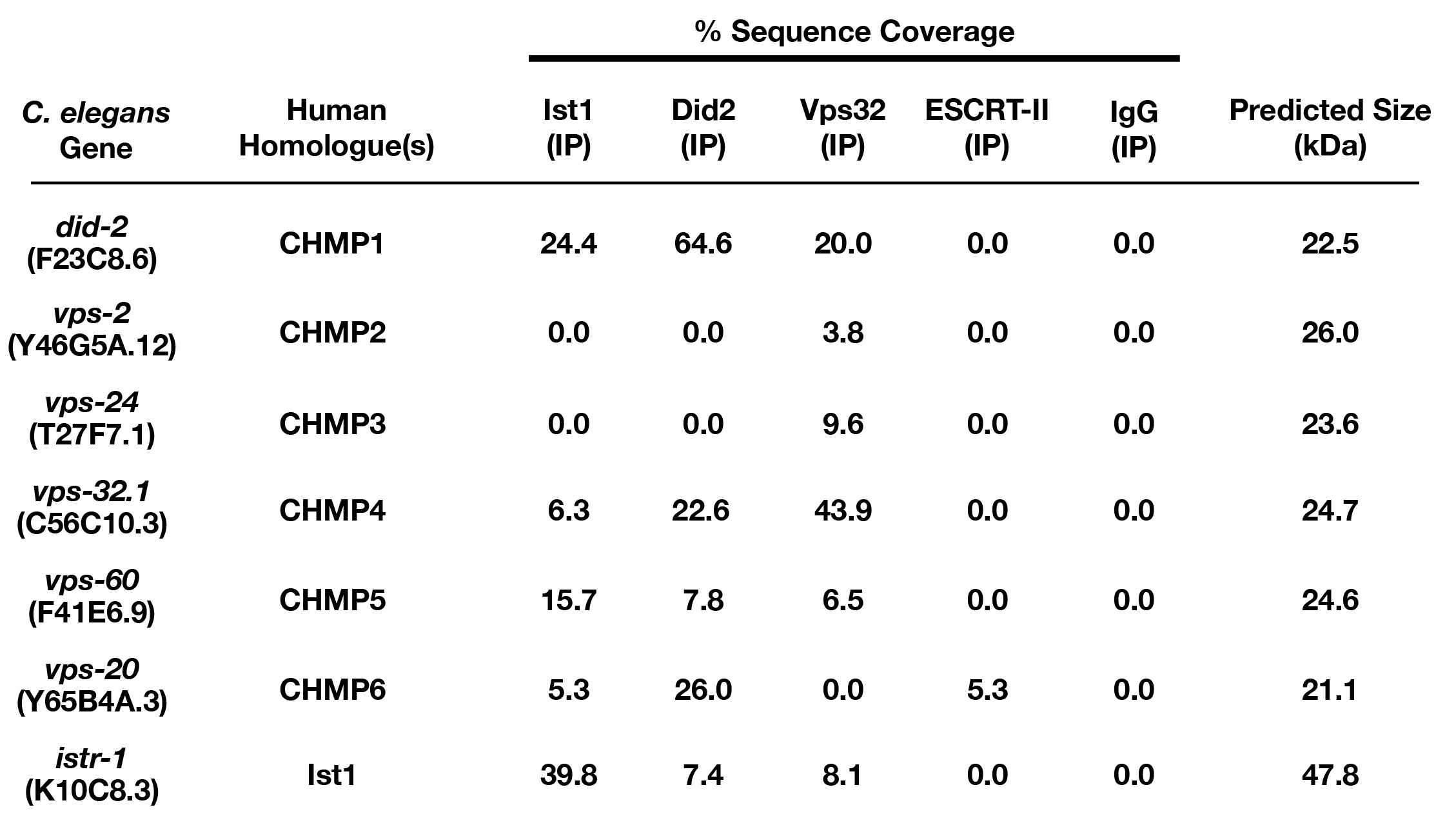

Supplement: Supplementary file 1 — Supplementary Information [file 41467_2017_1636_MOESM1_ESM.docx]
