# Supplementary material for: Ist1 regulates ESCRT-III assembly and function during multivesicular endosome biogenesis in Caenorhabditis elegans embryos
Source: Nat Commun. 2017 Nov 13;8:1439. doi: 10.1038/s41467-017-01636-8 (PMC5682282; doi:10.1038/s41467-017-01636-8)
Supplement: Supplementary file 3 — Description of Additional Supplementary Files [file 41467_2017_1636_MOESM3_ESM.pdf]

Description of Additional Supplementary Files

File Name: Supplementary Movie 1
Description: Rme2::GFP dynamics during ovulation and early embryo development. Time lapse confocal imaging was used to examine Rme2::GFP dynamics during oocyte ovulation and early embryogenesis.

File Name: Supplementary Movie 2
Description: GFP::Cav1 dynamics during ovulation and early embryo development. Time lapse confocal imaging was used to examine GFP::Cav1 and Histone H2B (mCherry::His58) dynamics during oocyte ovulation and early embryogenesis.

File Name: Supplementary Movie 3
Description: GFP::Cav1 dynamics during ovulation and early embryo development following Vps32 depletion. Time lapse confocal imaging was used to examine GFP::Cav1 dynamics during oocyte ovulation and early embryogenesis in the absence of Vps32.

File Name: Supplementary Movie 4
Description: Representative electron tomogram of wild type MVEs. High pressure frozen animals were processed for electron tomography to visualize newly formed MVEs within the one-cell stage embryo. Over 100 tomograms of wild type MVEs were collected. Bar, 100 nm.

File Name: Supplementary Movie 5
Description: Representative electron tomogram of MVE compartments in a Tsg101 depleted one-cell stage embryo. Following depletion of Tsg101, animals were high pressure frozen and processed for electron tomography. At least 10 tomograms of MVEs in the one-cell stage embryo were collected. Bar, 100 nm.

File Name: Supplementary Movie 6
Description: Representative electron tomogram of MVE compartments in a Vps20 depleted one-cell stage embryo. Following depletion of Vps20, animals were high pressure frozen and processed for electron tomography. 10 tomograms of MVEs in the one-cell stage embryo were collected. Bar, 100 nm.

File Name: Supplementary Movie 7
Description: Representative electron tomogram of MVE compartments in a Vps32 depleted one-cell stage embryo. Following depletion of Vps32, animals were high pressure frozen and processed for electron tomography. At least 10 tomograms of MVEs in the one-cell stage embryo were collected. Bar, 100 nm.

File Name: Supplementary Movie 8
Description: Representative electron tomogram of MVE compartments in a Ist1 depleted one-cell stage embryo. Following depletion of Ist1, animals were high pressure frozen and processed for electron tomography. 10 tomograms of MVEs in the one-cell stage embryo were collected. Bar, 100 nm.
